# Untreated bleeds in people with hemophilia A in a noninterventional study and intrapatient comparison after initiating emicizumab in HAVEN 1–3

**DOI:** 10.1002/rth2.12782

**Published:** 2022-09-13

**Authors:** Michael U. Callaghan, Elina Asikanius, Michaela Lehle, Johannes Oldenburg, Johnny Mahlangu, Marianne Uguen, Sammy Chebon, Rebecca Kruse‐Jarres, Víctor Jiménez‐Yuste, Midori Shima, Peter Trask, Christine L. Kempton, Craig M. Kessler, Gallia G. Levy, Flora Peyvandi

**Affiliations:** ^1^ Department of Pediatrics Central Michigan University Detroit Michigan USA; ^2^ Product Development, F. Hoffmann‐La Roche Ltd Basel Switzerland; ^3^ Institute of Experimental Haematology and Transfusion Medicine University of Bonn Bonn Germany; ^4^ Haemophilia Comprehensive Care Centre, Faculty of Health Sciences University of the Witwatersrand and NHLS Johannesburg South Africa; ^5^ PDB Biostatistics, F. Hoffmann‐La Roche Ltd Basel Switzerland; ^6^ University of Washington and Washington Center For Bleeding Disorders Seattle Washington USA; ^7^ Department of Hematology La Paz Hospital, Autónoma University Madrid Spain; ^8^ Department of Pediatrics Nara Medical University Kashihara Japan; ^9^ Patient Centered Outcomes Research, Genentech, Inc. South San Francisco California USA; ^10^ Department of Hematology and Medical Oncology and Hemophilia of Georgia Center for Bleeding & Clotting Disorders of Emory Emory University School of Medicine Atlanta Georgia USA; ^11^ The Division of Coagulation Georgetown University Hospital Washington District of Columbia USA; ^12^ Product Development, Genentech, Inc. South San Francisco California USA; ^13^ Fondazione IRCCS Ca' Granda Ospedale Maggiore Policlinico, Angelo Bianchi Bonomi Hemophilia and Thrombosis Center Milan Italy; ^14^ Department of Pathophysiology and Transplantation Università degli Studi di Milano Milan Italy; ^15^ Agios Pharmaceuticals Cambridge Massachusetts USA; ^16^ The Finnish Medicines Agency Helsinki Finland; ^17^ Spark Therapeutics, Inc. Philadelphia Pennsylvania USA

**Keywords:** bleeding, factor VIII, hemophilia A, hemostasis, prophylaxis

## Abstract

**Background:**

Bleeding in people with hemophilia A can be life threatening, and intra‐articular bleeds can result in joint damage. Most clinical studies focus on treated bleeds, while bleeds not treated with coagulation factor(s) (untreated bleeds) are underreported.

**Objectives:**

We assessed the incidence of untreated bleeds during a noninterventional study (NIS) wherein people with hemophilia A, with or without factor VIII (FVIII) inhibitors, were managed according to standard practice.

**Patients/Methods:**

Using the Bleed and Medication Questionnaire, we prospectively collected data from three cohorts: Cohort A, adults/adolescents (age ≥12 years) with FVIII inhibitors; Cohort B, children (aged <12 years) with FVIII inhibitors; Cohort C, adults/adolescents without FVIII inhibitors. Untreated bleeds were analyzed for site, frequency, and etiology of bleeding and compared with those during emicizumab prophylaxis in the same individuals after transferring to a Phase III HAVEN trial.

**Results:**

In the 221 participants enrolled in the NIS (Cohort A, *n* = 103; Cohort B, *n* = 24; Cohort C, *n* = 94), the incidence of untreated bleeds was approximately 40% of all bleeds in people with FVIII inhibitors and 26.2% in adolescents/adults without inhibitors. Approximately 70% of treated bleeds and approximately 54% of untreated bleeds in adults/adolescents were in joints. Untreated joint bleeds were less common (7.1%) in children. Overall, intra‐individual comparisons showed reduced treated/untreated bleeds following transition from standard to emicizumab prophylaxis.

**Conclusion:**

A significant proportion of bleeding events are untreated in people with hemophilia A. There is a need to further understand why bleeds remain untreated and to capture such events in clinical studies.

**Essentials**

- In many clinical studies on people with hemophilia A, only treated bleeds are reported.
- We documented type, location, and patterns of untreated bleeds in a noninterventional study.
- We found that a significant proportion of bleeds in people with hemophilia A remain untreated.
- Incidence of untreated bleeds should be captured in clinical trials.

## 1 INTRODUCTION

Hemophilia A is a bleeding disorder caused by a coagulation factor VIII (FVIII) deficiency.^1^ Prophylaxis of people with hemophilia A aims to prevent bleeds; however, breakthrough bleeds can still occur in spite of treatment.^2^, ^3^, ^4^

Annualized bleeding rate (ABR) is often used as the primary end point in clinical trials because the frequency and cumulative occurrence of bleeds is strongly correlated with long‐term joint function.^3^ However, data collection on bleeds is often connected with treatment in clinical studies,^5^ potentially resulting in underestimated ABRs if only treated bleeds are documented, with untreated bleeds remaining unrecorded. When bleeding rates among adults/adolescents with hemophilia A (with or without FVIII inhibitors) and children with FVIII inhibitors were investigated in an observational, noninterventional study (NIS),^6^, ^7^, ^8^ the Bleed and Medication Questionnaire (BMQ), which requires patients to record bleeds independent of treatment, was implemented to facilitate prospective data collection on both untreated and treated bleeds. Data collected using the BMQ highlighted the need for ongoing treatment for bleeding events in people with hemophilia A, especially those with FVIII inhibitors.^6^, ^7^, ^8^ However, treated bleeds only accounted for a portion of the reported bleeds. Therefore, a baseline of treated and untreated bleeds in individuals on standard FVIII or bypassing agent therapy (taken on demand or prophylactically) needs to be established to serve as a comparator for treatment‐related outcomes with nonfactor therapies, such as emicizumab, a bispecific, humanized, monoclonal antibody, which demonstrated highly efficacious bleed protection across the HAVEN clinical study program.^9^, ^10^, ^11^, ^12^

The present analysis addresses the shortage of data on the type, location, and patterns of untreated bleeds among populations of people with hemophilia A of different ages and FVIII inhibitor status, providing a better understanding of the bleeds that are left untreated and could potentially contribute to long‐term arthropathy and other chronic conditions associated with hemophilia A.

## 2 METHODS

### 2.1 Study design and participants

**FIGURE 1:**
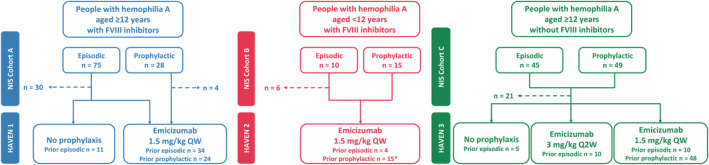
Disposition of participants in the NIS receiving episodic or prophylactic coagulation factor replacement and their transfer to the HAVEN Phase III clinical program investigating the safety and efficacy of emicizumab prophylaxis for hemophilia A. *One participant was not included in the intraindividual comparison due to a more stringent definition of what constituted prophylaxis compared with the HAVEN 2 primary analysis.^10^ The total number of participants enrolled to take episodic or prophylactic treatment regimens in the NIS was fixed, as defined by the study protocol.^6^, ^7^, ^8^ Abbreviations: FVIII, factor VIII; NIS, noninterventional study; Q2W, every 2 weeks; QW, every week

The NIS was a global prospective observational study collecting data on people with hemophilia A, with or without FVIII inhibitors, treated with the standard therapy at the time (Figure 1). Full details of the design of each study cohort have been published previously.^6^, ^7^, ^8^ Data from the NIS were used to assess the number of treated and untreated bleeds in a hemophilia A population that included children, adolescents, and adults, and to identify differences between populations with and without FVIII inhibitors.

In brief, the NIS enrolled participants from May 2015 to March 2017 at 33 sites in 12 countries.^6^, ^7^, ^8^ Cohort A comprised adults/adolescents (aged 12 years or older) with congenital hemophilia A (any severity) and high‐titer FVIII inhibitor history, episodic or prophylactic bypassing agent use for 6 months or more, and 6 or more or 2 or more bleeds in the past 6 months on episodic or prophylactic treatment, respectively.^7^ Cohort B comprised children (aged less than 12 years) with congenital hemophilia A, high‐titer FVIII inhibitor history, receiving either episodic or prophylactic bypassing agents, and 4 or more bleeds in the past 6 months (participants aged 2–11 years or older) or 2 or more bleeds in the previous 3 months (participants aged less than 2 years).^8^ Cohort C comprised adults/adolescents with severe congenital hemophilia A (FVIII activity less than 1%) and no history of FVIII inhibitors, and episodic or prophylactic FVIII use for 150 days or more before enrollment.^6^ Participants receiving episodic therapy must have had 5 or more bleeds in the past 6 months. Eligible participants from the NIS could subsequently enroll in one of the HAVEN trials; these were Phase III, open‐label studies evaluating emicizumab prophylaxis and had eligibility criteria similar to the NIS. Full details of the designs of the HAVEN studies have been previously published.^9^, ^10^, ^11^ Intraindividual comparisons were performed for participants who received bypassing agent or FVIII prophylaxis in the NIS and then emicizumab prophylaxis in a HAVEN study.

All studies were conducted in accordance with the International Conference on Harmonization guidelines for Good Clinical Practice and the principles of the Declaration of Helsinki. Study protocols were approved by the relevant independent ethics committee or institutional review board at each participating institution. All participants, or their legally authorized representatives for those aged less than 18 years, provided written informed consent before study participation. This NIS (ClinicalTrials.gov identifier: NCT02476942) and the randomized controlled interventional HAVEN 1 (NCT02622321), HAVEN 2 (NCT02795767), and HAVEN 3 (NCT02847637) studies were registered on ClinicalTrials.gov before the first patients were enrolled.

### 2.2 Data collection

Data from the NIS and HAVEN studies were used to assess the number of treated and untreated bleeds, differences in incidence between populations with and without FVIII inhibitors, and the impact of emicizumab therapy.

The BMQ was used to prospectively collect data on bleed occurrence and medication use. Information on bleeding and treatment were collected separately, enabling analysis of untreated versus treated bleeds. Using a hand‐held device, participants were prompted to report perceived bleeds and/or medication use via the BMQ at least once every 7 days.

Information recorded included the type, location, and cause of bleeds; and treatment information, including the timing, dose, and purpose of treatment. Further details of the questions the participants and caregivers responded to in the BMQ are provided in Table S1.

### 2.3 Data analyses

The NIS was not designed as a confirmatory study; all analyses were descriptive.^6^, ^7^, ^8^ Participants were followed up for efficacy (bleed‐related end points) and safety from the first day of reporting until study withdrawal or completion.^6^, ^7^, ^8^

Model‐based ABR was estimated via a negative binomial regression model, as used later in the HAVEN studies, which accounted for follow‐up times (efficacy periods) as an offset in the model. Individual patient ABRs were calculated using the equation: ABR = [(number of bleeds)/(number of days during efficacy period)] × 365.25. Model‐based ABRs (95% confidence interval [CI]) and calculated medians (interquartile range) of the individual participant ABRs are reported. The model‐based approach, which employed negative binomial regression and included the participant component in the model, was extended to intraindividual comparisons for participants in the NIS who transferred to a HAVEN study.

Analysis end points included treated bleeds, all bleeds, spontaneous bleeds, and joint bleeds. Untreated bleeds were investigated as a subset of all bleeds. In the NIS and the HAVEN studies, bleeds were recorded according to the ISTH Scientific and Standardization Committee (SSC) definitions,^13^ wherein bleeds of the same type and at the same anatomic location are counted as one bleed if the second bleed occurs within 72 h after stopping treatment for the first bleed (the “72‐hour rule”). As the 72‐hour rule would apply differently to untreated bleeds (i.e., the time period would be relative to the previous bleed rather than relative to previous treatment), it was not applied in the present analysis. As a result, each bleed was counted individually, allowing for clear identification and comparison of proportions of treated and untreated bleeds.

An event was considered a treated bleed if coagulation factors were administered and the participant specified on the BMQ that the reason for this was to treat a bleed (Table S1); this was irrespective of the time between the bleed and the treatment. A bleed and the first treatment thereafter were considered to be linked (i.e., one treatment belonged to one bleed only), with the following exception: If multiple bleeds occurred on the same calendar day, the subsequent treatment was considered to apply for each of these multiple bleeds. Any bleed at a different location was considered a separate bleed, regardless of time from the last treatment. Untreated bleeds were any bleeds not treated with coagulation factors; “all bleeds” comprised the sum of untreated and treated bleeds.

The NIS cohorts applied the joint bleed definition of their corresponding HAVEN study, with definitions varying slightly across the three trials. Cohort A and HAVEN 1 employed the ISTH SSC definition, whereby a bleed was defined by the sensation of an aura combined with another joint bleed symptom. The definition for Cohort C and HAVEN 3 differed only in that an aura was not required. For Cohort B and HAVEN 2, a joint bleed was defined on the basis of the location of the bleed reported as being a joint; because of their age (less than 12 years), these participants were not expected to identify symptoms or the sensation of an aura. These definitions were presented in the BMQ to aid their identification (Table S1).

While bleeds attributable to a surgery/procedure were not included in the NIS primary end point of treated bleeds,^6^, ^7^, ^8^ the purpose of the present study was to provide a comprehensive description of the nature of treated and untreated bleeds; therefore, all types of bleeds were included in all analyses, including those related to surgeries/procedures.

Data analyses were conducted by study statisticians and clinical pharmacologists (employed by the sponsor) who vouch for the completeness and accuracy of the statistical analyses. Data were made available to all authors, who confirm adherence to the protocol and statistical analyses plans.

## 3 RESULTS

### 3.1 Study population

In total, 221 participants were enrolled in the NIS. Cohort A (*n* = 103) were monitored for a median (range) of 26.0 (4.1–69.6) weeks. One participant withdrew for unknown reasons before reporting any bleed data and was not included in the analysis population. Cohort B (*n* = 24) were monitored for 23.4 (8.7–44.1) weeks, with all participants completing the study. Cohort C (*n* = 94) were monitored for 29.8 (12.4–47.7) weeks. Four participants in Cohort C discontinued prematurely (two were nonadherent, one was lost to follow‐up, and one died).

Participants were male, except for one female adult/adolescent participant with FVIII inhibitors (Table 1). Approximately one third of Cohort A, and half of Cohort B had previously been treated with immune tolerance induction therapy.

**TABLE 1:** Baseline demographics and characteristics of participants in the NIS^6^, ^7^, ^8^

Baseline characteristic	Cohort A: Adults/adolescents with FVIII inhibitors (*n* = 103)	Cohort B: Children with FVIII inhibitors (*n* = 24)	Cohort C: Adults/adolescents without FVIII inhibitors (*n* = 94)
Male, *n* (%)	102 (99.0)	24 (100)	94 (100)
Age, years, median (range)	31.0 (12–75)	7.5 (2–11)	34.0 (12–76)
Race, *n* (%)			
Asian	33 (32.0)	8 (33.3)	22 (23.4)
Black/African American	10 (9.7)	2 (8.3)	7 (7.4)
Caucasian	59 (57.3)	11 (45.8)	53 (56.4)
Multiple/unknown	1 (1.0)	3 (12.5)	12 (12.7)
Previously treated with immune tolerance induction, *n* (%)	32 (31.1)	12 (50.0)	NA
Efficacy and observation period, weeks, median (range)	26.0 (4.1–69.6)	23.4 (8.7–44.1)	29.8 (12.4–47.7)

Abbreviations: FVIII, factor VIII; NA, not applicable.

### 3.2 Incidence of untreated bleeds in the NIS

**FIGURE 2:**
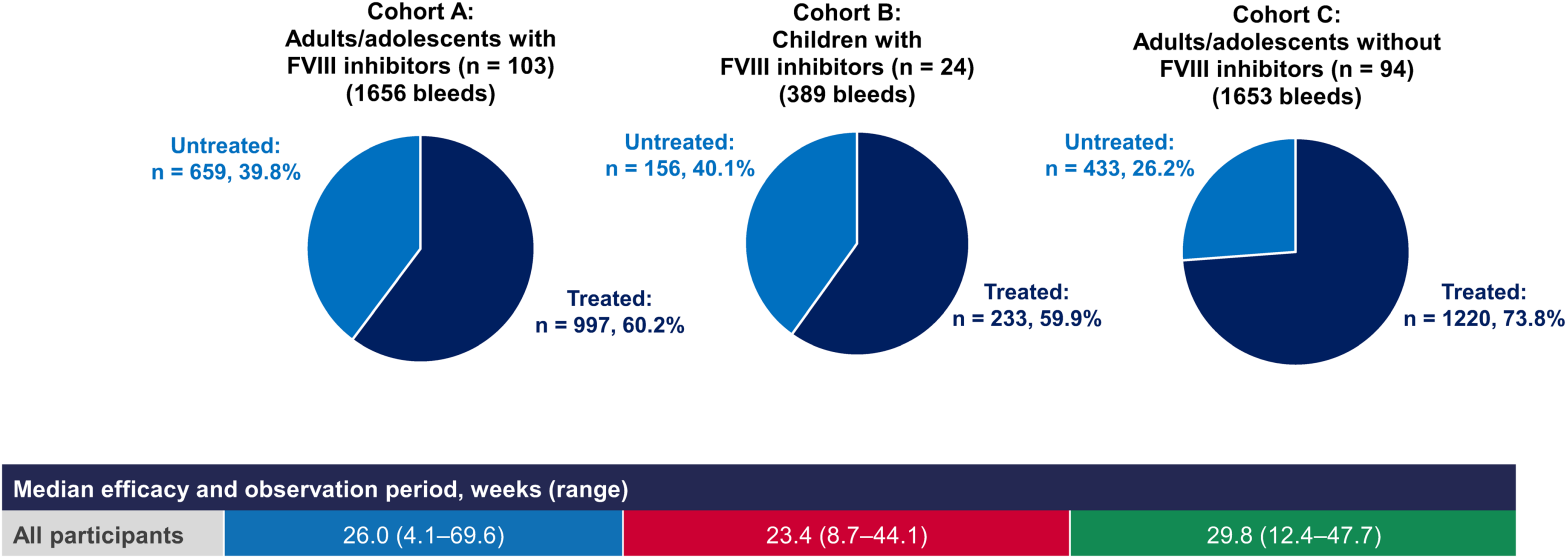
Proportion of treated* versus untreated bleeds among adult/adolescent and pediatric participants with or without FVIII inhibitors during the NIS. *For this analysis of treated bleeds, any symptoms of bleeding at the same location or locations with treatment administered ≤72 h apart, were *not* considered a single bleed and were counted separately. Abbreviations: FVIII, factor VIII; NIS, noninterventional study

The incidences of treated and untreated bleeds are displayed in Figure 2. In total, 659 untreated bleeds were observed in Cohort A, 156 in Cohort B, and 433 in Cohort C. The proportion of untreated bleeds was comparable between the cohorts with FVIII inhibitors, A and B (39.8% and 40.1% of all bleeds, respectively). In Cohort C, however, 26.2% of bleeds were untreated. Untreated bleeds were reported by 71.8% (74/103) of participants in Cohort A, 54.2% (13/24) of those in Cohort B, and 47.9% (45/94) of those in Cohort C.

**FIGURE 3:**
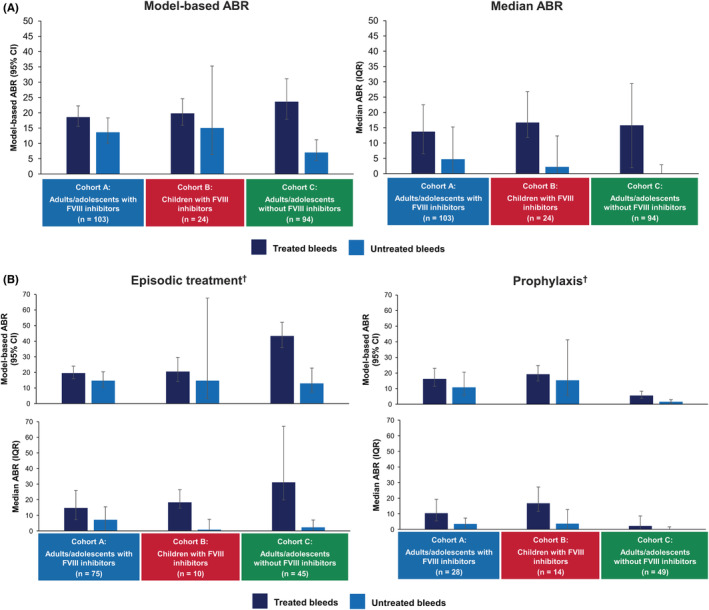
ABRs for treated* and untreated bleeds in the three cohorts overall and according to whether participants received episodic treatment or prophylaxis. (A) Model‐based and calculated median ABRs for treated and untreated bleeds in adult/adolescent participants with FVIII inhibitors, pediatric participants with FVIII inhibitors and adult/adolescent participants without FVIII inhibitors; (B) model‐based and calculated median ABRs for treated and untreated bleeds for episodic treatment or prophylaxis among adult/adolescent and pediatric participants with or without FVIII inhibitors.^†^ *For this analysis of treated bleeds, any symptoms of bleeding at the same location or locations with treatment administered ≤72 h apart, were *not* considered a single bleed and were counted separately. ^†^Episodic and prophylactic treatments refer to bypassing agents in individuals with FVIII inhibitors and FVIII in individuals without FVIII inhibitors. Abbreviations: ABR, annualized bleeding rate; CI, confidence interval; FVIII, factor VIII; IQR, interquartile range

The model‐based ABR (95% CI) for untreated bleeds was similar for Cohort A (13.6 [10.06–18.35]) and Cohort B (15.0 [6.36–35.31]), but notably lower for Cohort C (7.0 [4.33–11.20]) (Figure 3A). This difference appeared to be mainly driven by the low ABR for the group of participants in Cohort C receiving prophylactic therapy (1.5 [0.75–2.93]), with the ABR for those receiving episodic therapy being higher, at 12.9 (7.35–22.75) (Figure 3B).

For participants with FVIII inhibitors who had undergone immune tolerance induction therapy, the model‐based ABR (95% CI) was lower for adults/adolescents (6.2 [3.49–11.11]) compared with children (16.3 [4.63–57.50]) (Table S2).

Approximately 50% of the untreated bleeds in the participants receiving prophylactic treatment in each cohort were followed within 24 hours by a dose of coagulation factor recorded by the participants as being for prophylaxis (Table S3).

### 3.3 Untreated bleeds in the NIS: Locations and causes

For the adult/adolescent cohorts, A and C, the majority of both treated and untreated bleeds were reported in joints (54.0%–70.8%) (Table 2). The untreated bleeds were distributed among the knee (18.4%–28.7%), elbow (21.0%–27.8%), ankle (15.7%–30.8%), and other locations (23.0%–34.6%). In Cohort B, however, only 7.1% of untreated bleeds were in joints, with these most commonly located in the knee (63.6%).

**TABLE 2:** Locations of treated and untreated bleeds in the NIS

Location	Treated bleeds^a^ (%)			Untreated bleeds (%)		
	Cohort A: Adults/adolescents with FVIII inhibitors (*n* = 103)	Cohort B: Children with FVIII inhibitors (*n* = 24)	Cohort C: Adults/adolescents without VIII inhibitors (*n* = 94)	Cohort A: Adults/adolescents with FVIII inhibitors (*n* = 103)	Cohort B: Children with FVIII inhibitors (*n* = 24)	Cohort C: Adults/adolescents without FVIII inhibitors (*n* = 94)
Total number of bleeds, *n*	997	233	1220	659	156	433
Joint, *n* (%)^b^	706 (70.8)	118 (50.6)	856 (70.2)	362 (54.9)	11 (7.1)	234 (54.0)
Knee	226 (32.0)	21 (17.8)	196 (22.9)	104 (28.7)	7 (63.6)	43 (18.4)
Elbow	166 (23.5)	48 (40.7)	261 (30.5)	76 (21.0)	1 (9.1)	65 (27.8)
Ankle	149 (21.1)	31 (26.3)	227 (26.5)	57 (15.7)	1 (9.1)	72 (30.8)
Other^c^	165 (23.4)	18 (15.2)	172 (20.1)	125 (34.6)	2 (18.2)	54 (23.0)
Muscle, *n* (%)^b^	152 (15.2)	38 (16.3)	220 (18.0)	89 (13.5)	12 (7.7)	141 (32.6)
Soft tissue,^d^ *n* (%)^b^	58 (5.8)	NA	NA	72 (10.9)	NA	NA
Bruise/hematoma,^d^ *n* (%)^b^	39 (3.9)	NA	NA	104 (15.8)	NA	NA
Miscellaneous,^d^ *n* (%)^b^	42 (4.2)	NA	NA	32 (4.9)	NA	NA
Other,^e^ *n* (%)^b^	0 (0)	77 (33.0)	144 (11.8)	0 (0)	133 (85.3)	58 (13.4)

Abbreviations: BMQ, Bleed and Medications Questionnaire; FVIII, factor VIII; NA, not applicable.

^a^ For this analysis of treated bleeds, any symptoms of bleeding at the same location or locations with treatment administered ≤72 h apart, were *not* considered a single bleed and were counted separately.

^b^ *n* refers to number of bleeds and % refers to percentage of total bleeds.

^c^ Includes wrist, fingers/thumb, shoulder, hip and toes.

^d^ In addition to joint and muscle bleeds, participants in Cohort A (adults/adolescents with FVIII inhibitors) had the option to record bleed types as soft tissue bleeds, bruise/hematoma, or miscellaneous bleeds, whereas for Cohorts B (pediatrics with FVIII inhibitors) and C (adults/adolescents without FVIII inhibitors), these additional categories were substituted by the bleed type “other.” This was as a result of a change in the BMQ used.

^e^ Bleeds in joint locations such as the knee, ankle or elbow were occasionally reported under the bleed type “other,” for example, when the bleed was around the joint (such as bruises or hematomas).

In contrast with treated muscle bleeds, the proportions of which were similar across the three cohorts (15.2%–18.0%), untreated muscle bleeds varied in frequency, with proportions of 13.5% and 32.6% for adults/adolescents with and without inhibitors, respectively, and 7.7% for the pediatric cohort (Table 2). The predominant location for untreated bleeds in pediatric participants was “other” (85.3%); these bleeds were most commonly in the knee, mouth, back of knee, or shin. This differed from treated bleeds, of which only 33.0% were in this category.

**FIGURE 4:**
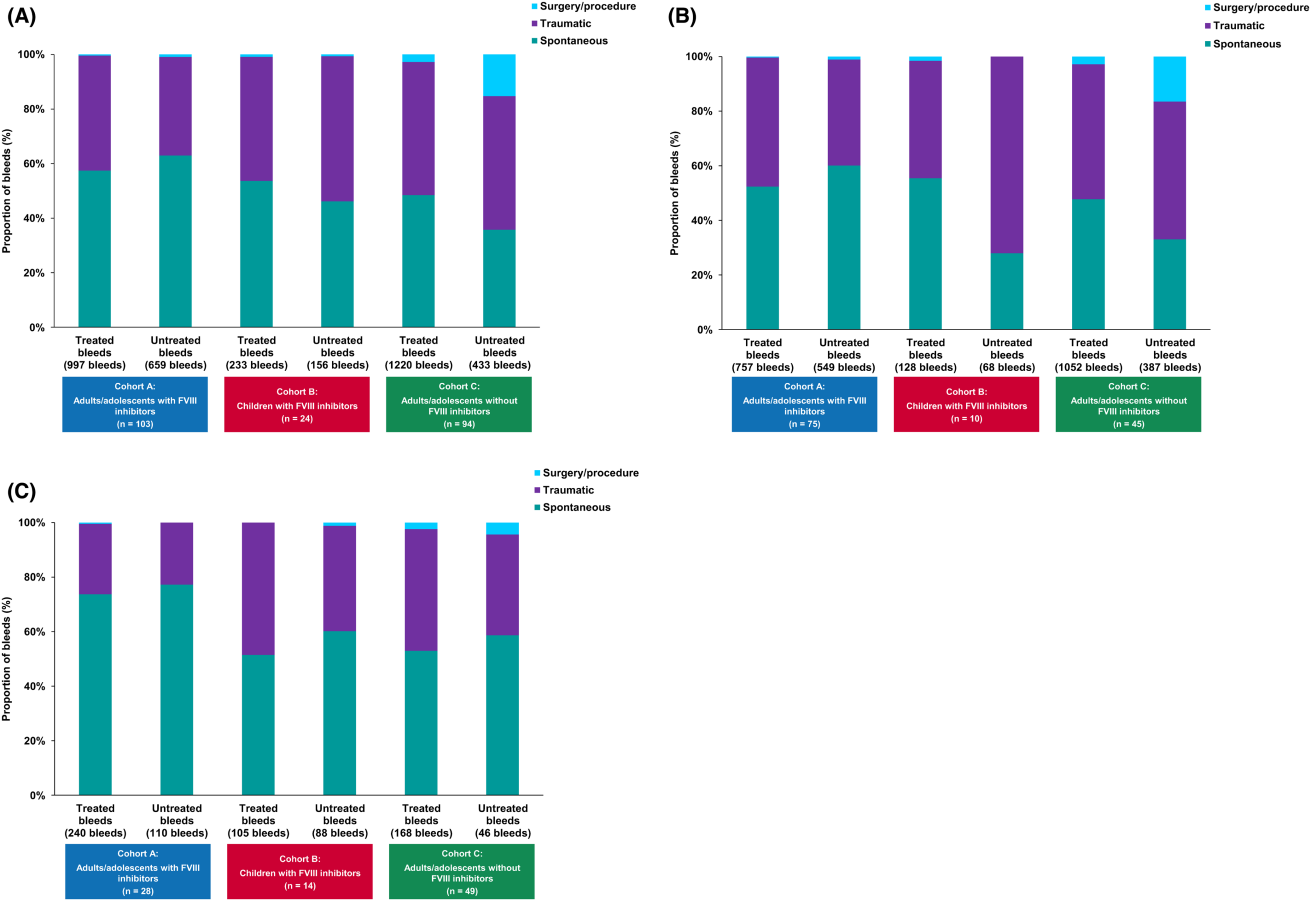
Causes of treated* and untreated bleeds in the three cohorts overall and according to whether participants received episodic treatment or prophylaxis. (A) Cause of treated and untreated bleeds; (B) cause of treated and untreated bleeds in participants administered episodic therapy^†^; (C) cause of treated and untreated bleeds in participants administered prophylactic therapy. *For this analysis of treated bleeds, any symptoms of bleeding at the same location or locations with treatment administered ≤72 h apart, were *not* considered a single bleed and were counted separately. ^†^Episodic and prophylactic treatments refer to bypassing agents in individuals with FVIII inhibitors and FVIII in individuals without FVIII inhibitors. Abbreviations: FVIII, factor VIII

Spontaneous bleeds accounted for 63.0% of untreated bleeds in adults/adolescents with FVIII inhibitors and 35.8% in those without FVIII inhibitors (Figure 4A). Surgery/procedural untreated bleeds accounted for a lower proportion of untreated bleeds in adults/adolescents with FVIII inhibitors versus those without FVIII inhibitors (0.6%–0.9% vs. 15.2%).

Causes of bleeding in participants receiving episodic therapy were generally comparable with those for the overall population, except for the increased proportion of traumatic untreated bleeds in children (72.1% vs. 53.2% in the overall pediatric cohort; Figure 4B). In general, higher proportions of untreated bleeds were spontaneous among participants receiving prophylaxis (58.7%–77.3%; Figure 4C) versus those taking episodic treatment (27.9%–60.1%; Figure 4B).

### 3.4 Intraindividual comparison of bleeds in the NIS versus following enrollment in the emicizumab interventional studies

Among participants receiving prophylaxis in the NIS, 24 adults/adolescents with FVIII inhibitors entered the HAVEN 1 study, 14 children with FVIII inhibitors entered the HAVEN 2 study, and 48 adults/adolescents without FVIII inhibitors entered the HAVEN 3 study. All transferred participants received emicizumab 1.5 mg/kg every week in their respective HAVEN study.

**FIGURE 5:**
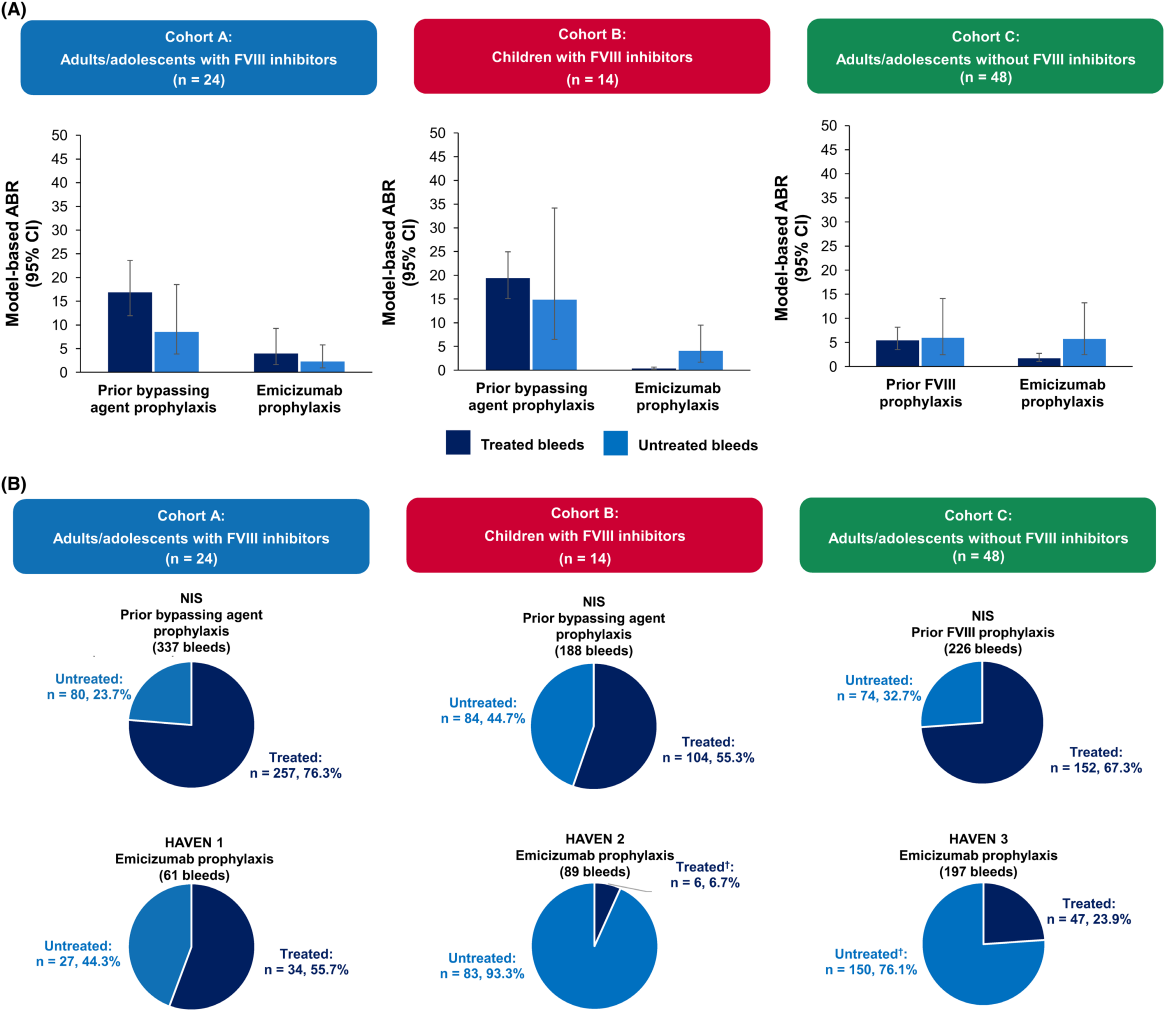
Intraindividual comparisons of treated* and untreated bleeds for participants in the NIS who then transferred onto the corresponding HAVEN study. (A) Intraindividual comparisons of model‐based ABRs among participants who had previously enrolled in the NIS (prior FVIII/bypassing agent) and were switched to a HAVEN study (emicizumab); (B) intraindividual comparisons of treated versus untreated bleeds among participants who had previously enrolled in the NIS (prior FVIII/bypassing agent) and were switched to weekly emicizumab in one of the HAVEN studies. *For this analysis of treated bleeds, any symptoms of bleeding at the same location or locations with treatment administered ≤72 h apart, were *not* considered a single bleed and were counted separately. Data include bleeds attributable to surgery/procedure. ^†^Bleeds attributable to surgeries/procedures accounted for <7% of bleeds in each category (Table 3), with the exception of treated bleeds in HAVEN 2 (1/6 bleeds; 16.7%) and untreated bleeds in HAVEN 3 (67/150 bleeds; 44.7%). Abbreviations: ABR, annualized bleeding rate; CI, confidence interval; FVIII, factor VIII; NIS, noninterventional study

For adults/adolescents with FVIII inhibitors, the ABR (95% CI) for untreated bleeds decreased from 8.5 (3.86–18.54) on prophylaxis with a bypassing agent in the NIS to 2.3 (0.95–5.79) on emicizumab in HAVEN 1, while for pediatric participants, it decreased from 14.8 (6.44–34.16) in the NIS to 4.0 (1.67–9.50) in HAVEN 2 (Figure 5A). No change was seen in untreated bleeds in adults/adolescents without FVIII inhibitors taking prophylaxis (5.9 [2.43–14.12] for FVIII in the NIS versus 5.7 [2.47–13.22] for emicizumab in HAVEN 3).

The proportions of bleeds that were untreated were higher in each of the HAVEN studies than in their corresponding NIS cohort (Figure 5B). The proportions of both treated and untreated bleeds that were spontaneous decreased for participants taking emicizumab compared with previous standard prophylaxis in the NIS (Table 3). There was a notable increase in the proportion of untreated bleeds associated with surgeries/procedures in HAVEN 3 compared with Cohort C in the NIS (44.7% vs. 2.7%); however, the majority of these bleeds occurred in a single participant. This individual underwent a high number of percutaneous drainage tube procedures because of a historical peritoneocutaneous fistula, which resulted in a high number of muscle bleeds. Sensitivity analysis demonstrated similar proportions of untreated bleeds related to surgeries/procedures in the NIS and HAVEN 3 when this participant was excluded (3.2% vs. 2.4%; Table S4). Furthermore, the ABR (95% CI) for untreated bleeds in HAVEN 3 decreased from 5.7 (2.47–13.22) with the participant included to 2.8 (1.30–6.03) when excluded (Figure S1).

**TABLE 3:** Types and locations of treated and untreated bleeds in individuals treated with standard prophylaxis in the NIS followed by emicizumab in HAVEN 1–3

	Treated bleeds^a^ (%)						Untreated bleeds (%)					
	Cohort A: Adults/adolescents with FVIII inhibitors		Cohort B: Children with FVIII inhibitors		Cohort C: Adults/adolescents without FVIII inhibitors		Cohort A: Adults/adolescents with FVIII inhibitors		Cohort B: Children with FVIII inhibitors		Cohort C: Adults/adolescents without FVIII inhibitors	
Prophylaxis	Bypassing agent (*n* = 24)	Emicizumab (*n* = 24)	Bypassing agent (*n* = 14)	Emicizumab (*n* = 14)	FVIII (*n* = 48)	Emicizumab (*n* = 48)	Bypassing agent (*n* = 24)	Emicizumab (*n* = 24)	Bypassing agent (*n* = 14)	Emicizumab (*n* = 14)	FVIII (*n* = 48)	Emicizumab (*n* = 48)
Total number of bleeds, *n*	257	34	104	6	152	47	80	27	84	83	74	150
Type, *n* (%)^b^												
Spontaneous	201 (78.2)	22 (64.7)	55 (52.9)	0 (0)	74 (48.7)	15 (31.9)	62 (77.5)	12 (44.4)	49 (58.3)	16 (19.3)	37 (50.0)	19 (12.7)
Traumatic	55 (21.4)	10 (29.4)	49 (47.1)	5 (83.3)	74 (48.7)	29 (61.7)	18 (22.5)	15 (55.6)	34 (40.5)	66 (79.5)	35 (47.3)	64 (42.7)
Surgery	1 (0.4)	2 (5.9)	0 (0)	1 (16.7)	4 (2.6)	3 (6.4)	0 (0)	0 (0)	1 (1.2)	1 (1.2)	2 (2.7)	67 (44.7)
Location												
Joint, *n* (%)^b^	185 (72.0)	24 (70.6)	51 (49.0)	3 (50.0)	88 (57.9)	27 (57.4)	38 (47.5)	13 (48.1)	5 (6.0)	5 (6.0)	45 (60.8)	21 (14.0)
Knee	57 (30.8)	6 (25.0)	12 (23.5)	1 (33.3)	18 (20.5)	10 (37.0)	6 (15.8)	4 (30.8)	4 (80.0)	2 (40.0)	12 (26.7)	5 (23.8)
Elbow	31 (16.8)	6 (25.0)	18 (35.3)	0 (0)	36 (40.9)	4 (14.8)	3 (7.9)	2 (15.4)	0 (0)	0 (0)	9 (20.0)	1 (4.8)
Ankle	57 (30.8)	8 (33.3)	12 (23.5)	2 (66.7)	25 (28.4)	10 (37.0)	14 (36.8)	3 (23.1)	0 (0)	3 (60.0)	13 (28.9)	13 (61.9)
Other^c^	40 (21.6)	4 (16.7)	9 (17.7)	0 (0)	9 (10.2)	3 (11.1)	15 (39.5)	4 (30.7)	1 (20.0)	0 (0)	11 (24.4)	2 (9.5)
Muscle, *n* (%)^b^	42 (16.3)	6 (17.6)	21 (20.2)	1 (16.7)	23 (15.1)	6 (12.8)	8 (10.0)	2 (7.4)	3 (3.6)	1 (1.2)	16 (21.6)	74 (49.3)
Soft tissue,^d^ *n* (%)^b^	14 (5.4)	0 (0)	NA	NA	NA	NA	6 (7.5)	0 (0)	NA	NA	NA	NA
Bruise/hematoma,^d^ *n* (%)^b^	10 (3.9)	0 (0)	NA	NA	NA	NA	23 (28.8)	0 (0)	NA	NA	NA	NA
Miscellaneous,^d^ *n* (%)^a^	6 (2.3)	0 (0)	NA	NA	NA	NA	5 (6.3)	0 (0)	NA	NA	NA	NA
Other,^e^ *n* (%)^b^	0 (0)	4 (11.8)	32 (30.8)	2 (33.3)	41 (27.0)	14 (29.8)	0 (0)	12 (44.4)	76 (90.5)	77 (92.8)	13 (17.6)	55 (36.7)

Abbreviations: BMQ, Bleed and Medications Questionnaire; FVIII, factor VIII; NA, not applicable.

^a^ For this analysis of treated bleeds, any symptoms of bleeding at the same location or locations with treatment administered ≤72 h apart, were *not* considered a single bleed and were counted separately.

^b^ *n* refers to number of bleeds and % refers to percentage of total bleeds.

^c^ Includes wrist, fingers/thumb, shoulder, hip and toes.

^d^ In addition to joint and muscle bleeds, participants in Cohort A (adults/adolescents with FVIII inhibitors) had the option to record bleed types as soft tissue bleeds, bruise/hematoma, or miscellaneous bleeds, whereas for Cohorts B (pediatrics with FVIII inhibitors) and C (adults/adolescents without FVIII inhibitors), these additional categories were substituted by the bleed type “other.” This was as a result of a change in the BMQ used.

^e^ Bleeds in joint locations such as the knee, ankle, or elbow were occasionally reported under the bleed type “other,” for example, when the bleed was around the joint (such as bruises or hematomas).

The majority of treated bleeds observed in the three cohorts in the NIS and during HAVEN 1–3 were located in joints (49.0%–72.0%) (Table 3). The locations of untreated bleeds, however, varied between cohorts. High proportions of adults/adolescents with FVIII inhibitors had untreated joint bleeds in both the NIS and HAVEN 1 (47.5% and 48.1%, respectively) (Table 3). For adults/adolescents without FVIII inhibitors, emicizumab was associated with a lower proportion of participants with untreated joint bleeds than was FVIII prophylaxis (14.0% vs. 60.8%), but higher percentages in muscle (49.3% vs. 21.6%) and other bleeds (36.7% vs. 17.6%). Untreated joint bleeds were less common in children than in adults.

## 4 DISCUSSION

This study quantitatively demonstrates the high proportion of bleeds that remain untreated in people with hemophilia A (26%–40% of total bleeds).

Untreated bleeds can contribute significantly to arthropathy and disease burden in people with hemophilia A.^14^ Yet joint bleeds can be difficult to diagnose, and it is often hard to distinguish bleed symptoms from those of existing arthropathy. There is a need for better understanding of the types, locations, and patterns and severity of bleeds that are not treated. Collecting data on these untreated bleeds is therefore clinically important; however, many clinical studies document only treated bleeds.^5^ In this study, the BMQ allowed participants to capture bleeds and bleed‐related treatment independently, providing granular information on the relative incidence of treated and untreated bleeds. This allows a more comprehensive and informative evaluation of therapies.

Untreated bleeds were more common in people with FVIII inhibitors compared with those without FVIII inhibitors. The reason for a greater proportion of bleeds remaining untreated in individuals with FVIII inhibitors remains unknown but could be related to a perceived lack of efficacy or unpredictability of bypassing agents, treatment burden, or reduced access to treatment.^15^, ^16^, ^17^, ^18^, ^19^ In adults with prior immune tolerance induction therapy, however, there was a lower ABR for untreated bleeds (6.2 [3.49–11.11]) compared with the whole adult population with FVIII inhibitors (13.6 [10.06–18.35]). We hypothesize that people with hemophilia A with FVIII inhibitors who have previously received immune tolerance induction are already accustomed to administering frequent treatments, so may be more willing to treat bleeds.

Joints were the most common locations for untreated bleeds in the adult/adolescent cohorts. In contrast, for pediatric participants, only a small proportion of untreated bleeds were located in the joints, with the rest recorded as soft tissue, bruise/hematoma, or miscellaneous. This notable difference between adults and children may be linked to the decision to treat being made by the caregiver as opposed to the child themselves. It may also be attributable to the difficulty in differentiating joint pain from bleeding, which would affect the adults with preexisting arthropathy significantly more than children with more pristine joints. Future studies should examine treatment decision‐making behavior among people with hemophilia A and caregivers, and the long‐term sequelae associated with untreated bleeds.

Intraindividual comparisons showed that ABRs for untreated bleeds decreased for people with hemophilia A of all ages with FVIII inhibitors when they transitioned from the NIS to the respective HAVEN study, but remained fairly constant for those without FVIII inhibitors. Overall, the reduced number of treated and untreated bleeds with emicizumab provides an indication of the effect of treatment on cumulative bleeding, an important consideration in managing people with hemophilia A. The risk of progressive joint and muscle damage, leading to loss of motion, arthropathy, muscle atrophy, pain, and contractures, does not discriminate between treated and untreated bleeds^20^; therefore, it is important to reduce all bleeds.^2^

The NIS and HAVEN 1–3 studies were limited by the BMQ relying on patient reporting of bleeds, which could result in both under‐/overreporting of events and a lack of interindividual standardization. Additionally, the specific location of bleeds outside of joints and muscles were classified differently for the cohorts, meaning that these data could not be compared between groups. The finding that approximately half of the participants who were on prophylaxis in the NIS administered a dose of factor concentrate within 24 h after an untreated bleed adds further complexity to the interpretation of these data. It is feasible that, outside the confines of a clinical trial setting, these individuals may have simply adjusted their prophylaxis dosing schedule as needed to address breakthrough bleeds, but it is not possible to confirm this definitively with the data from this analysis, particularly given that many of those on prophylaxis with short‐acting FVIII or bypassing agents would administer their treatment every 1–3 days regardless of bleeding events. Further, this does not account for the remaining cases in which bleeds were left untreated despite the next dose of prophylaxis not being due for more than 24 h.

The perceived degree of bleed severity and physician instructions regarding bleed treatment may influence individual treatment decisions, limiting the interpretation of change in untreated bleeds from the NIS to HAVEN studies. Bleeds may have also been managed differently during the HAVEN studies compared with standard treatment in a noninterventional setting. Furthermore, participants' reason(s) for not treating bleeds were not investigated, limiting conclusions regarding behaviors influencing bleed treatment. The intraindividual comparison, however, offered a robust design controlling for participant‐related confounders in an otherwise descriptive study, which is a strength of this analysis.

## 5 CONCLUSIONS

In conclusion, a significant proportion of subjectively perceived bleeds in people with hemophilia A remain untreated, suggesting that the full burden of the disease is not adequately captured in many clinical studies. The decision to treat a bleed or not is complex and may be influenced by patient‐specific factors such as age and the presence of an inhibitor, as well as bleed and treatment characteristics. An understanding of why some bleeding events remain untreated is needed, and capturing these events in clinical trials would provide a more comprehensive evaluation of therapies.

## AUTHOR CONTRIBUTIONS

M. U. Callaghan, J. Oldenburg, M. Shima, R. Kruse‐Jarres, J. Mahlangu, V. Jiménez‐Yuste, and F. Peyvandi contributed to the study design, collected data for this study, and contributed to the interpretation of the data. M. Lehle, E. Asikanius, M. Uguen, P. Trask, C. M. Kessler, and G. G. Levy contributed to the study design and participated in both the analysis and interpretation of the data. S. Chebon and C. L. Kempton participated in both the analysis and interpretation of the data. All authors critically reviewed the manuscript, approved the final manuscript as submitted, and agree to be accountable for all aspects of the work.

## FUNDING INFORMATION

The study was funded by F. Hoffmann‐La Roche Ltd and Chugai Pharmaceutical Co., Ltd.

## RELATIONSHIP DISCLOSURE

MUC has received consultancy fees from F. Hoffmann‐La Roche Ltd/Genentech, Inc., Takeda, Pfizer, Sanofi, Kedrion, Uniqure, Spark Therapeutics, BioMarin, Hema Biologics, Global Blood Therapeutics, Bluebird Bio, Catalyst, Chiesi, Bayer, Emmaus, and has received speaker's bureau fees from Takeda, Novo Nordisk, F. Hoffmann‐La Roche Ltd/Genentech, Inc., Global Blood Therapeutics, BioMarin, and Bayer. EA is a previous employee of F. Hoffmann‐La Roche Ltd. ML is an employee and holds stocks in F. Hoffmann‐La Roche Ltd; JO has received personal/consultancy fees for travel support, advisory boards, and symposia from Bayer, Biogen Idec, BioMarin, Biotest, Chugai Pharmaceutical Co., Ltd, CSL Behring, Freeline, Grifols, Novo Nordisk, Octapharma, Pfizer, F. Hoffmann‐La Roche Ltd, Sanofi, Spark Therapeutics, SOBI, and Shire/Takeda; and grants from Bayer, Biotest, CSL Behring, Octapharma, and Pfizer. JM has received research funding from BioMarin, CSL Behring, Novo Nordisk, SOBI, F. Hoffmann‐La Roche Ltd and Uniqure; is a member of advisory committees for Baxalta, CSL Behring, Catalyst Biosciences, Novo Nordisk, F. Hoffmann‐La Roche Ltd and Spark Therapeutics; and has received speaker's bureau fees from Novo Nordisk, Pfizer, SOBI, Shire, F. Hoffmann‐La Roche Ltd, ISTH, and (World Federation of Hemophilia) WFH. MU is an employee of F. Hoffmann‐La Roche Ltd; SC is an employee and holds stocks in F. Hoffmann‐La Roche Ltd. R.K.‐J. has received honoraria and consultancy fees from Genentech, Inc./F. Hoffmann‐La Roche Ltd, CSL Behring, BioMarin, and CRISPR; has been a member of the speaker's bureau for Genentech, Inc./F. Hoffmann‐La Roche Ltd and Sanofi; and has received research funding from Genentech, Inc. VJ‐Y has received reimbursement for attending symposia/congresses, honoraria for speaking, consulting, and/or research funding from Takeda, Bayer, CSL Behring, Grifols, Novo Nordisk, SOBI, F. Hoffmann‐La Roche Ltd, Octapharma, BioMarin, Sanofi, and Pfizer. MS has received research funding from Chugai Pharmaceutical Co., Ltd, Sanofi, Takeda, CSL Behring, KM Biologics Co., Ltd., and Novo Nordisk; consulting fees from Chugai Pharmaceutical Co., Ltd, and Fujimoto Pharmaceutical Corporation; and speaker's bureau fees from Chugai Pharmaceutical Co., Ltd, Sanofi, Bayer AG, and Sysmex; is listed as an entity's board of directors or advisory board committee member for Chugai Pharmaceutical Co., Ltd, F. Hoffmann‐La Roche Ltd, BioMarin, Bayer AG, and Sanofi; and is an inventor of patents related to anti‐FIXa/FX bispecific antibodies. PT is an employee and holds stocks in F. Hoffmann‐La Roche Ltd/Genentech, Inc. CLK has received honoraria for participation in advisory boards for Sanofi US, Takeda, and Spark Therapeutics. CMK has received research support from Bayer, Genentech, Inc., Novo Nordisk, Octapharma, and Takeda; and has served on advisory boards for Bayer, CSL Behring, Genentech, Inc. Novo Nordisk, Octapharma, Takeda, Pfizer, and HEMA Biologics. GGL is an employee of Spark Therapeutics and holds shares in F. Hoffmann‐La Roche Ltd. FP has received speaker fees for participating in educational symposia and advisory boards for F. Hoffmann‐La Roche Ltd, Sanofi, SOBI, and Takeda.

## Supporting information


Appendix S1

## Data Availability

Qualified researchers may request access to individual patient‐level data through the clinical study data request platform (https://vivli.org/). Further details on Roche's criteria for eligible studies are available at https://vivli.org/members/ourmembers/. For further details on Roche's Global Policy on the Sharing of Clinical Information and how to request access to related clinical study documents, see https://www.roche.com/research_and_development/who_we_are_how_we_work/clinical_trials/our_commitment_to_data_sharing.htm.
