# Supplementary material for: Untreated bleeds in people with hemophilia A in a noninterventional study and intrapatient comparison after initiating emicizumab in HAVEN 1–3
Source: Res Pract Thromb Haemost. 2022 Sep 13;6(6):e12782. doi: 10.1002/rth2.12782 (PMC9468791; doi:10.1002/rth2.12782)
Supplement: Supplementary file 1 — Appendix S1 [file RTH2-6-e12782-s001.pdf]

SUPPORTING INFORMATION

Untreated bleeds in a noninterventional study of people with hemophilia A and intrapatient comparison after emicizumab initiation in HAVEN 1–3

Contents

- Additional data comparisons
- **Table S1**: Descriptions of how data were collected in the Bleed and Medication Diaries for the NIS
- **Table S2**: ABRs for treated and untreated bleeds in adults/adolescents and children with hemophilia A with FVIII inhibitors who had undergone immune tolerance induction in the NIS.
- **Table S3**: Time between bleeds and subsequent hemophilia medication
- **Table S4**: Sensitivity analysis showing intra-individual comparison of untreated bleeds in participants in Cohort C of the NIS who transferred to HAVEN 3, with and without one participant who experienced 65 untreated bleeds associated with surgeries in HAVEN 3
- **Figure S1**. Sensitivity analysis showing intra-individual comparison of ABRs for treated and untreated bleeds in participants in Cohort C of the NIS who transferred to HAVEN 3, with (A) and without (B) one participant who experienced 65 untreated bleeds associated with surgeries during HAVEN 3

Additional data comparisons

- Annualized bleed rates (ABRs; 95% CI) for treated bleeds were similar across cohorts, whether considering adults/adolescents with FVIII inhibitors (18.6 [15.59–22.26]) versus children with FVIII inhibitors (19.8 [15.90–24.63]) or adults with FVIII inhibitors versus those without (23.6 [17.86–31.12]) (Fig. 3A).
- ABRs (95% CI) for untreated bleeds in participants with FVIII inhibitors (13.6 [10.06–18.35]) were higher than for participants without FVIII inhibitors (7.0 [4.33–11.20]) (Fig. 3A).
- The magnitude of the difference in ABRs (95% CI) for adults/adolescents with FVIII inhibitors receiving episodic versus prophylactic therapy for treated bleeds (19.5 [15.90–24.01] vs 16.2

[11.40–23.01]) was relatively low, at 3.3 (Fig. 3B). A similar trend was seen with untreated bleeds, where the difference was 3.8 (14.6 [10.48–20.45] vs 10.8 [5.62–20.62]) (Fig. 3B).

- The magnitude of the difference in ABRs (95% CI) for adults/adolescents without FVIII inhibitors receiving episodic versus prophylactic therapy for treated bleeds was relatively high, at 37.8 (43.3 [35.99–52.07] vs 5.5 [3.65–8.41]) (Fig. 3B). The difference was 11.4 for untreated bleeds (12.9 [7.35–22.75] vs 1.5 [0.78–2.93]) (Fig. 3B).
- ABRs (95% CI) were lower for adults/adolescents with FVIII inhibitors administered episodic therapy compared with participants without FVIII inhibitors (19.5 [15.90–24.01] vs 43.3 [35.99–52.07]) for treated bleeds, but the ABRs for untreated bleeds were comparable (14.6 [10.48–20.45] vs 12.9 [7.35–22.75]).
- With prophylactic therapy, the ABR (95% CI) for treated bleeds was 16.2 (11.40–23.01) for adults/adolescents with FVIII inhibitors versus 5.5 (3.65–8.41) for those without inhibitors, while the ABRs (95% CI) for untreated bleeds in the same respective populations were 10.8 (5.62–20.62) versus 1.5 (0.78–2.93) (Fig. 3B).

TABLE S1. Descriptions of how data were collected in the Bleed and Medication Diaries for the NIS

**Cohort**	**Bleed cause**	**Bleed location***	**Medication administered**
**Cohort A:**<br><br>**Adults/adolescents with FVIII inhibitors**	Participants were asked if they had undergone a procedure/surgery or injured themselves, participated in strenuous activity, or overused a joint/muscle that they believed may have caused the bleed.	Participants were asked for the location of their bleed, with the options being joint, muscle, soft tissue, bruise/hematoma, or miscellaneous. In cases where joint or muscle bleeds were selected, an additional step encouraged them to select, from a list, the symptoms that led to their choice.	Participants were asked to record any administration of hemophilia medication. They were asked to report the purpose of the medication, with options being 'usual prophylactic dose', 'one-time prophylactic dose', 'treatment for a bleed', or for a 'procedure/surgery'. They were also asked to link any medication given to treat a bleed with the specific bleed that had been previously recorded.
**Cohort B:**<br><br>**Children with FVIII inhibitors**	Caregivers of pediatric participants were asked to report when the bleed started and if the child had undergone a procedure/surgery or injured themselves, participated in strenuous activity, or overused a joint/muscle that the caregiver believed may have caused the bleed.	Caregivers were asked to record the location of the bleed, with the options being joint, muscle, or other. Definitions were provided to aid with the identification of joint and muscle bleeds.	Caregivers were asked to record any administration of hemophilia medication. In addition to recording the product, time, and dose, they were asked to report the purpose of the medication, with options being 'usual prophylactic dose', 'treatment for a bleed', 'preventive dose before activity', or 'preventive dose for procedure/surgery'.
**Cohort C:**<br><br>**Adults/adolescents without FVIII inhibitors**	Participants were asked if they had undergone a procedure/surgery or injured themselves, participated in strenuous activity, or overused a joint/muscle that they believed may have caused the bleed.	Participants were asked for the location of their bleed, with the options being joint, muscle, or other. In cases where joint or muscle bleeds were selected, an additional step encouraged them to select, from a list, the symptoms that led to their choice.	Participants were asked to record any administration of hemophilia medication. In addition to recording the type, time, and dose, they were asked to report the purpose of the medication, with options being 'usual prophylactic dose', 'treatment for a bleed', 'preventive dose before activity', or 'preventive dose for procedure/surgery'.

*The lists of symptoms for joint/muscle bleeds were based on the ISTH SSC definitions (Blanchette et al. J Thromb Haemostas 2014).

NIS, non-interventional study

Table S2. ABRs for treated and untreated bleeds in adults/adolescents and children with hemophilia A with FVIII inhibitors who had undergone immune tolerance induction in the NIS

	Treated bleeds		Untreated bleeds	
	**Adults/ adolescents with FVIII inhibitors (n = 32)**	**Children with FVIII inhibitors (n = 12)**	**Adults/ adolescents with FVIII inhibitors (n = 32)**	**Children with FVIII inhibitors (n = 12)**
Model-based ABR (95% CI)	21.6 (14.94–31.08)	22.2 (17.61–28.09)	6.2 (3.49–11.11)	16.3 (4.63–57.50)
Calculated median ABR (IQR)	13.2 (5.60–26.09)	21.2 (12.88–30.23)	2.0 (0.00–6.15)	1.4 (0.00–29.27)

ABR, annualized bleeding rate; CI, confidence interval; FVIII, factor VIII; IQR, interquartile range; NIS, noninterventional study.

Table S3. Time* between bleeds and subsequent hemophilia medication

	Cohort A: Adults/adolescents with FVIII inhibitors (n = 103)		Cohort B: Children with FVIII inhibitors (n = 24)		Cohort C: Adults/adolescents without FVIII inhibitors (n = 94)	
	Episodic bypassing agent (n = 75)	Prophylactic bypassing agent (n = 28)	Episodic bypassing agent (n = 10)	Prophylactic bypassing agent (n = 14)	Episodic FVIII (n = 45)	Prophylactic FVIII (n = 49)
**Treated bleeds**						
Number of treated bleeds followed by medication	713§	221	121	101	871¶	151
Time between bleed and medication, n (%)†						
0–24 hours	638 (89.5)	202 (91.4)	109 (90.1)	97 (96.0)	805 (92.4)	141 (93.4)
24–48 hours	47 (6.6)	13 (5.9)	9 (7.4)	3 (3.0)	26 (3.0)	7 (4.6)
48–72 hours	14 (2.0)	4 (1.8)	1 (0.8)	0	9 (1.0)	2 (1.3)
>72 hours	14 (2.0)	2 (0.9)	2 (1.7)	1 (1.0)	31 (3.6)	1 (0.7)
**Untreated bleeds**						
Number of untreated bleeds	531	104	64	85	208	39
Number of untreated bleeds followed by prophylaxis‡	188**	96††	0	85	17‡‡	38††
Time between bleed and prophylaxis, n (%)						

0–24 hours	31 (16.5)	47 (49.0)	0	55 (64.7)	14 (82.4)	19 (50.0)
24–48 hours	6 (3.2)	22 (22.9)	0	15 (17.6)	2 (11.8)	12 (31.6)
48–72 hours	6 (3.2)	10 (10.4)	0	8 (9.4)	0	1 (2.6)
>72 hours	145 (77.1)	17 (17.7)	0	7 (8.2)	1 (5.9)	6 (15.8)

*For multiple bleeds within a 72-hour period at the same location, the time was calculated from the start of the first bleed.

†Where reason for medication was given as 'treatment for bleed' in the BMQ.

‡Where reason for medication was given as 'prophylaxis' in the BMQ.

§For 3 treated bleeds, the time from bleed to medication could not be derived because either the exact time of bleed or medication was missing.

¶For 1 treated bleed, the time from bleed to medication could not be derived because either the exact time of bleed or medication was missing.

**Some participants who were receiving episodic treatment at the start of the study reported administering "one-time prophylaxis" (n=35) or "usual prophylactic dose" (n=6) at some point during the study period. Out of the 6 patients that reported "usual prophylactic dose", 3 patients switched to prophylactic treatment, 1 patient received usual prophylaxis doses for 2 months after a road traffic accident, and 2 patients received a single prophylactic dose most likely reported as "usual prophylactic dose" by mistake.

††In some cases, the next dose of prophylaxis following a bleed was not captured as the bleed occurred near to the end of the study and so the prophylaxis was administered outside of the efficacy period.

‡‡ Three participants who were receiving episodic treatment at the start of the study reported administering prophylaxis at some point during the study period, with all of these having switched to prophylactic treatment during the study.

BMQ, bleed and medication questionnaire; FVIII, factor VIII

Table S4. Sensitivity analysis showing intra-individual comparison of untreated bleeds in participants in Cohort C of the NIS who transferred to HAVEN 3, with and without one participant who experienced 65 untreated bleeds associated with surgeries in HAVEN 3*

	**Including participant**		**Excluding participant**	
	**FVIII prophylaxis (n = 48)**	**Emicizumab prophylaxis (n = 48)**	**FVIII prophylaxis (n = 47)**	**Emicizumab prophylaxis (n = 47)**
Total number of untreated bleeds, n	74	150	62	83
Type of bleed, n (%)				
Spontaneous	37 (50.0)	19 (12.7)	27 (43.5)	17 (20.5)
Traumatic	35 (47.3)	64 (42.7)	33 (53.2)	64 (77.1)
Surgery	2 (2.7)	67 (44.7)	2 (3.2)	2 (2.4)

*This participant also reported a high number of untreated bleeds associated with surgery in the NIS; however, they were being treated with episodic FVIII at the time and so are not included in the present analysis. The participant changed to prophylactic FVIII during the study, and experienced no bleeds due to surgery when on this treatment regimen (30 days in duration).

FVIII, factor VIII; NIS, non-interventional study

Figure S1. Sensitivity analysis showing intra-individual comparison of ABRs for treated and untreated bleeds in participants in Cohort C of the NIS who transferred to HAVEN 3, with (A) and without (B) one participant who experienced 65 untreated bleeds associated with surgeries during HAVEN 3*

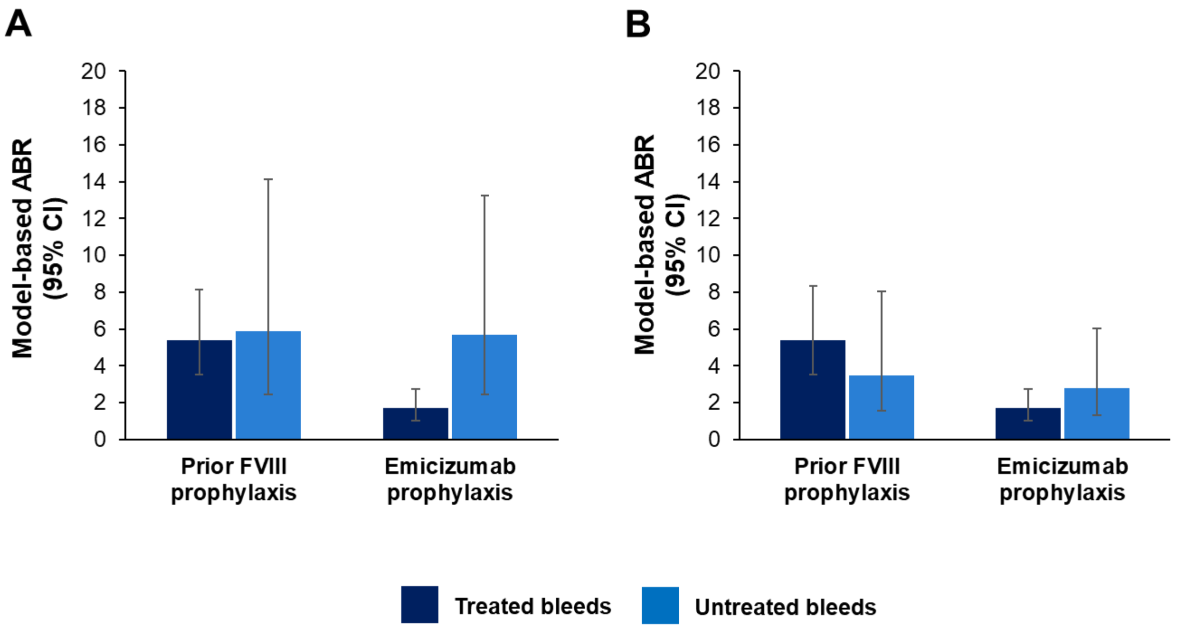

*This participant also reported a high number of untreated bleeds associated with surgery in the NIS; however, they were being treated with episodic FVIII at the time and so are not included in the present analysis.

ABR, annualized bleeding rate; CI, confidence interval; FVIII, factor VIII; NIS, non-interventional study.
